# A Survey on Depressive Symptoms and Its Correlates Amongst Physicians in Bangladesh During the COVID-19 Pandemic

**DOI:** 10.3389/fpsyg.2022.846889

**Published:** 2022-07-26

**Authors:** M. Tasdik Hasan, Afifa Anjum, Md. Abdullah Al Jubayer Biswas, Sahadat Hossain, Sayma Islam Alin, Kamrun Nahar Koly, Farhana Safa, Syeda Fatema Alam, Md. Abdur Rafi, Vivek Podder, Md. Moynul Hossain, Tonima Islam Trisa, Dewan Tasnia Azad, Rhedeya Nury Nodi, Fatema Ashraf, S. M. Quamrul Akther, Helal Uddin Ahmed, Roisin McNaney

**Affiliations:** ^1^Public Health Foundation, Bangladesh (PHF, BD), Dhaka, Bangladesh; ^2^Action Lab, Department of Human Centred Computing, Faculty of Information Technology, Monash University, Melbourne, VIC, Australia; ^3^Department of Primary Care and Mental Health, University of Liverpool, Liverpool, United Kingdom; ^4^Department of Public Health and Informatics, Jahangirnagar University, Dhaka, Bangladesh; ^5^Department of Public Health, State University of Bangladesh, Dhaka, Bangladesh; ^6^International Centre for Diarrheal Diseases Research, Bangladesh (icddr, b), Dhaka, Bangladesh; ^7^Department of Public Health, Dalla Lana School of Public Health, University of Toronto, Toronto, ON, Canada; ^8^Shaheed Suhrawardy Medical College Hospital, Dhaka, Bangladesh; ^9^Rajshahi Medical College, Rajshahi, Bangladesh; ^10^Tairunnessa Memorial Medical College and Hospital, Gazipur, Bangladesh; ^11^Department of Public Health, North South University, Dhaka, Bangladesh; ^12^Jashore Medical College, Jashore, Bangladesh; ^13^National Institute of Mental Health, Dhaka, Bangladesh

**Keywords:** COVID-19, Bangladesh, physicians, depression symptoms, PHQ-9

## Abstract

**Aim:**

The aim of this study was to determine the presence of depressive symptoms and understand the potential factors associated with these symptoms among physicians in Bangladesh during the COVID-19 pandemic.

**Methods:**

A cross-sectional study using an online survey was conducted in between April 21 and May 10, 2020, among physicians living in Bangladesh. Participants completed a series of demographic questions, COVID-19-related questions, and the Patient Health Questionnaire-9 (PHQ-9). Descriptive statistics (frequency, percentage, mean and standard deviation), test statistics (chi-squared test and logistic regression) were performed to explore the association between physicians’ experience of depression symptoms and other study variables. Stepwise binary logistic regression was followed while conducting the multivariable analysis.

**Result:**

A total of 390 physicians completed the survey. Of them, 283 (72.6%) were found to be experiencing depressive symptoms. Predictors which were significantly associated with depressive symptoms were gender (with females more likely to experience depression than males), the presence of sleep disturbance, being highly exposed to media coverage about the pandemic, and fear around (a) COVID-19 infection, (b) being assaulted/humiliated by regulatory forces and (c) by the general public, while traveling to and from the hospital and treating patients during the countrywide lockdown.

**Conclusion:**

The findings of this study demonstrate that there is a high prevalence of depressive symptom among physicians especially among female physicians in Bangladesh during the COVID-19 pandemic. Immediate, adequate and effective interventions addressing gender specific needs are required amid this ongoing crisis and beyond.

## Introduction

The COVID-19 pandemic has caused unparalleled levels of fear, anxiety, and uncertainty globally (Dai et al., 2020). The spread of this virus presents a global health threat with catastrophic effects on every aspect of human life (Gallopeni et al., 2020). During this pandemic, paying close attention to the mental health of practicing physicians is even more so important, as they are encountering severe health risks to themselves and their loved ones (Asmundson and Taylor, 2020). Physicians working within the COVID-19 pandemic have been put under immense pressure as they contend with multiple factors, including stressful working hours, lack of personal protective equipment (PPE), lack of adequate medications and necessary hospital logistics (i.e., oxygen supply and high flow nasal cannula), and being away from their families (Lai et al., 2020; Rafi et al., 2021). Some contemporary studies conducted in different parts of the world have found that fear of becoming infected, as well as spreading the virus to family and friends, resulted in high levels of symptoms of stress, depression, and anxiety among physicians and other health care workers (Lee et al., 2007; Cabarkapa et al., 2020; Tasnim et al., 2021). For example, a cross-sectional study on health care providers in India found high-level stress and depressive symptoms among participants (Wilson et al., 2020). Sahin et al. (2020) observed similar findings in their study of Turkish health care workers, with 939 participants (Sahin et al., 2020).

In Bangladesh, the first COVID-19 case was identified on 08 March 2020 (Hasan et al., 2020). By 25 May, 2022 there were 1,953,298 confirmed cases and the total death toll was 29,130 in the country (DGHS, 2022). The total number of affected physicians was 3,182, with a tragic loss of 190 physicians by 22 February, 2022 (BMA, 2022). Infections among physicians rolled out across the country due to a lack of safe health service infrastructure, including insufficient monitoring of health guidelines and a lack of required safety equipment, e.g., PPE (Rafi et al., 2021).

The COVID-19 pandemic has revealed enormous gaps within the healthcare system of Bangladesh (Al-Zaman, 2020). In addition, mental health is historically one of the most neglected areas of public health research (Hasan et al., 2021a,b). Given that Bangladesh is a low resource setting with often inadequate healthcare facilities, dealing with both COVID-19 and the psychological issues that have emerged due to the outbreak is even more challenging. Despite this, there have been very little studies regarding mental health of physicians. Barua et al. (2020) investigated psychological burden of the pandemic among the frontline doctors and they used PHQ-4 to determine anxiety & depression (Barua et al., 2020). Tasnim et al. (2021) reported anxiety and depression among the frontline health works including data from doctors, nurses, public health professionals, lab workers and other caregivers (Tasnim et al., 2021); Khatun et al. (2021) reported findings on mental health of physicians though the sample size was very low (Khatun et al., 2021) to reach to a conclusion; Debnath et al. (2021) investigated with a very limited sample (Debnath et al., 2021) of intern doctors; and Hasan et al. (2022) reported anxiety and depression among physician’s using HADS scale. These critical findings indicated a high prevalence rate of depression symptoms/clinical depression and they call for additional reports to portray the gravity of the situation. Indeed, there is still a crucial need to explore the jolt of COVID-19 on Bangladeshi physicians, to uncover the impact that this pandemic has had on their mental health in relation to gender and other relevant socio-cultural factors to support them adequately during public health crisis like this.

In this study, we aim to determine the severity of depressive symptoms among physicians in Bangladesh, using a widely acceptable tool, the PHQ-9, and examine the potential predictors associated with the pandemic.

## Materials and Methods

### Study Design

A cross-sectional online survey was conducted in between 21 April to 10 May 2020, on Bangladesh Medical & Dental Council (BM&DC) registered physicians, when the COVID-19 pandemic and the enforced zonal lockdown was in its initial phase in the country. Considering the contemporary situation and risks associated with face-to-face data collection in hospital settings amid the pandemic, the survey was web based. An English version survey questionnaire was designed on a Google form, so that respondents could access it from any device with an internet connection. An open survey invitation was sent to potential respondents *via* email or social network includes personal Facebook account, twitter profile, etc. (see further details below).

### Sampling Technique and Procedure

The sample size was calculated using a single population proportion formula, considering the prevalence of depression among healthcare workers as 22.8%. As no study reported rates of depression among physicians in Bangladesh while we started the investigation, we used a pooled prevalence of depression among healthcare workers as estimated by a meta-analysis during the pandemic situation for our sample size determination (Cabarkapa et al., 2020). We applied the following assumptions: 95% confidence level, 5% absolute precision, 1.27 design effect, 10% non-response rate, which yielded a total sample of size 378. A systematic sampling technique was used for recruiting respondents to the study. A contact list of 2,340 physicians was prepared using the professional networks of the research team, including potential participants’ email or social network includes personal Facebook account, twitter profile, etc. Five volunteers (young physicians) from the authors’ institutions were employed to send out invitations to participate using the contact. The invitation contained a brief background and the objectives of the study, as well as information related to maintaining confidentiality and the link to the survey that would be used. Physicians who accepted the invitation were able to access the web survey the link to provide their response. If the physician rejected the invitation the research team approached another physician according to their position on the contact list. The research team approached a total of 585 physicians (every fourth physician of the contact list). Of these, 157 physicians declined to take part in the study (all female) and 38 physician provided inconsistent or missing data. This left a total of 390 fully completed surveys for analysis. Data were anonymized at the point of collection (no personal data were linked to the survey). Only the volunteers who made initial contact knew the final list of physicians who agreed to participate.

### Ethical Consideration

Informed consent was obtained using a tick box on the Google form. The study was conducted following the Checklist for Reporting Results of Internet E Surveys (CHERRIES) guideline (Eysenbach, 2004). It was strictly maintained by the authors to ensure that all procedures relevant to this work complied with the proper ethical standards of the Helsinki Declaration of 1975, as revised in 2008. The study was approved by the Ethical Review Committee, Shaheed Suhrawardy Medical College, Dhaka, Bangladesh (ShSMC/Ethical/2020/12).

### Data Collection Tool

The web survey had three parts: (i) demographic questions (age, gender, marital status, financial stability and adequacy of food), (ii) COVID19-related questions (the risk of exposure to COVID-19, access to PPE, any training they had been given about COVID-19, exposure to media, mental health history, and the psychological impacts of COVID 19 pandemic), and (iii) Patient-Health Questionnaire-9 (PHQ-9).

### Measures

Depression (outcome): For the identification of depressive symptom as well as the severity level of it among the participants, a well-established, popularly used 9-item scale, PHQ-9 had been utilized in this study. The PHQ-9 is a commonly used tool to measure depression symptoms. It consists of nine diagnostic items which measures how often individuals have been bothered by certain depression risk factors over the past 2 weeks (e.g., little pleasure in doing things). Each item is scored on a scale of zero (not at all) to three (nearly every day). The global summation of the nine items represents the total PHQ-9 score which can be used to measure severity: minimal (0–4), mild (5-9), moderate (10-14), moderately severe (15-19), severe (20-27; Kroenke et al., 2001).

### Independent Variable(s)

These variables were—(a) Socio-demographic characteristics (respondent’s socio demographic characteristics: gender, age and marital status); (b) Household related information (data on whether the household, the respondent is residing in, had financial solvency and had enough food during the period of data collection); (c) COVID-19 susceptibility (participants’ susceptibility to COVID-19 including having symptoms of COVID-19, being exposed to COVID-19 patients, treating any patient who was later tested COVID-19 positive and staying in quarantine); (d) Personal protective equipment (PPE; having sufficient PPE supply as well as the source from which PPE was received); (e) Training about COVID-19 (whether the participants were trained regarding COVID-19 management and the source of their training); (f) Exposed to media (frequency to check news update every day, got information to stay away from media, source of news and lastly hard to stay away from social media recently); (g) Respondent’s history of mental health (participant’s history of mental health problem and if they were taking any treatment or not at the moment of data collection were included in the survey); (h) The psychological attributes of COVID 19 pandemic [tension of getting infected, tension for a family member being infected by COVID-19, working hours (daily) before the pandemic, having sleeping disturbances, average sleeping hours in the last 4 weeks, agitated more quickly than usually and agitation about human contact]; and (i) Community impact of COVID 19 pandemic (facing any obstacles/humiliation while getting into or back from work by regulatory forces as well as fear of getting assaulted/humiliated on the way to the hospital or home).

### Statistical Analysis

Descriptive statistics were computed by using frequency (percentage for categorical variables) and mean and standard deviation (SD; for continuous variables) to describe the characteristics of the physicians. A cross-tabulation with Pearson’s chi-squared (*χ*^2^) test was then computed to examine the association between physicians’ experiences of depression and the factors of the study sample: Household related information, COVID-19 susceptibility, media exposure and others. A bivariable logistic regression analysis was then used to estimate the pairwise association of our outcome of interest (depression among physician) with explanatory variables, for instance, relationship between depression and the psychological attributes of COVID 19 pandemic. This was reported as unadjusted odds ratio with 95% confidence interval. Finally, multivariable logistic regression was exploited to explore the adjusted effect of explanatory variables on the physician depression and was reported as adjusted odds ratio with 95% confidence interval. During multivariable analysis, the stepwise algorithm was applied to find out potential factors associated with physician depression (Duan et al., 2020; Zhuo et al., 2020). Statistical significance level was set at value of *p* <0.05 and 95% confidence interval (CI). Data were analyzed using Stata software (Stata Corp. 2017. Stata Statistical Software: Release 15. College Station, TX: Stata Corp LP.).

## Results

### Socio-Demographic Characteristics

Characteristics of the study participants are shown in Table 1. The majority of the respondents were female (56.4%), aged between 24 and 27 years (59.5%), were unmarried (55.4%), and had sufficient earnings (45.9%) and food supply at their homes (63.0%).

**Table 1:** Prevalence of depression symptoms, assessed by PHQ-9, according to study participants different socio-demographic characteristics (*N* = 390).

Variables	Percentage (number)	Prevalence of depression			
		Number	percentage	95% CI	*p*-value
Among all physician	390	283	72.6	(23.2–32.1)	
Socio-demographic characteristics					
Age in year (Mean ± SD)	27.5 ± 4.4				
≤23	4.4 (17)	11	64.7	(39.6, 83.7)	
24–27	59.5 (232)	178	76.7	(70.8, 81.7)	0.112
28–31	26.4 (103)	71	68.9	(59.3, 77.2)	
≥32	9.7 (38)	23	60.5	(44.2, 74.8)	
Gender					
Male	43.6 (170)	106	62.4	(54.8, 69.4)	<0.001
Female	56.4 (220)	177	80.5	(74.6, 85.2)	
Marital status					
Married	42.6 (166)	108	65.1	(57.5, 72.0)	
Unmarried	55.4 (216)	167	77.3	(71.2, 82.4)	0.006
Divorced/separate	2.0 (8)	8	100.0		
Household related information					
Having sufficient earning					
Yes	45.9 (179)	116	64.8	(57.5, 71.5)	
No	36.4 (142)	121	85.2	(78.3, 90.2)	<0.001
Not sure	17.7 (69)	46	66.7	(54.7, 76.8)	
Sufficient supply of food at home					
Yes	63.0 (246)	178	72.4	(66.4, 77.6)	
No	10.3 (40)	34	85.0	(70.1, 93.2)	0.130
Not sure	26.7 (104)	71	68.3	(58.7, 76.5)	
COVID-19 susceptibility					
Having symptoms of COVID-19					
Yes	5.9 (23)	20	86.9	(65.8, 95.9)	
No	88.7 (346)	246	71.1	(66.1, 75.7)	0.173
May be	5.4 (21)	17	81.0	(58.1, 92.9)	
Exposed by COVID – 19 patients					
Yes	9.2 (36)	25	69.4	(52.5, 82.4)	0.660
No	90.8 (354)	258	72.9	(68.0, 77.3)	
Treating any patient who was later tested COVID-19 positive					
Yes	6.7 (26)	18	69.2	(49.0, 84.1)	
No	46.9 (183)	134	73.2	(66.3, 79.2)	0.910
Not sure	46.4 (181)	131	72.4	(65.4, 78.4)	
Staying in quarantine					
Yes	11.5 (45)	33	73.3	(58.5, 84.3)	
No	22.8 (89)	67	75.3	(65.2, 83.2)	0.781
Not applicable	65.6 (256)	183	71.5	(65.6, 76.7)	
Personal protective equipment (PPE)					
Getting enough PPE					
Yes	9.5 (37)	22	59.5	(42.9, 74.1)	
Got it but not enough in number	41.5 (162)	119	72.5	(66.1, 79.7)	0.292
Got it but not qualified enough to protect me	30.8 (120)	88	73.3	(64.7, 80.5)	
No	18.2 (71)	54	76.1	(64.7, 84.6)	
Source of PPE					
Self-funded	31.0 (121)	98	81.0	(72.9, 87.1)	
Government	4.6 (18)	12	66.7	(42.1, 84.6)	
Hospital	20.8 (81)	58	71.6	(60.8, 80.4)	0.035
Both Gob and hospital	14.6 (57)	35	61.4	(48.1, 73.2)	
Local people/NGOs/sponsored	10.8 (42)	26	61.9	(46.3, 75.4)	
Training about COVID-19					
Sufficiency of training about COVID-19					
Yes, from Government	3.3 (13)	9	69.2	(39.7, 88.5)	
Yes, from hospital	3.6 (14)	11	78.6	(49.3, 93.3)	
Yes, I took some online courses	26.9 (105)	74	70.5	(61.0, 78.5)	
I had some demonstration from my colleagues	5.4 (21)	17	80.9	(58.1, 92.9)	0.850
I am not sure, I read some contents	54.9 (214)	157	73.4	(67.0, 78.9)	
No	5.9 (23)	15	65.2	(43.7, 81.9)	
Exposed to media					
Frequency to check news update everyday					
One time	14.9 (58)	40	68.9	(55.9, 79.6)	
Two times	19.2 (75)	49	65.3	(53.8, 75.3)	
Three times	18.5 (72)	58	80.6	(69.7, 88.2)	0.365
Four times	4.3 (17)	12	70.6	(44.9, 87.6)	
Five times	2.8 (2.8)	7	63.6	(32.5, 86.4)	
More than five times	40.3 (157)	117	74.5	(67.1, 80.8)	
Got information to stay away from media					
Yes	51.8 (202)	167	82.7	(76.8, 87.3)	
No	33.3 (130)	75	57.7	(49.0, 65.9)	<0.001
Not sure	14.9 (58)	41	70.7	(57.6, 81.0)	
Source of news					
TV news (Online on Fb page, Youtube)	62.3 (243)	180	74.1	(68.2, 79.2)	
Newspaper (Online/offline)	3.3 (13)	6	46.2	(21.5, 72.8)	
News websites	3.6 (14)	9	64.3	(36.6, 84.9)	
International organization’s website	3.1 (12)	7	58.3	(29.6, 82.3)	0.062
Social Media	26.4 (103)	79	76.7	(67.5, 83.9)	
Govt. website	1.3 (5)	2	40.0	(8.2, 83.2)	
Hard to stay away from social media recently					
Yes, I spend more time than before on social media	52.6 (205)	158	77.1	(70.8, 82.3)	
Yes, but I did that previously too	13.1 (51)	34	66.7	(52.6, 78.3)	0.061
No, I do not find it hard	28.5 (111)	72	64.9	(55.5, 73.2)	
I do not get enough time to spend on	5.9 (23)	19	82.6	(61.1, 93.5)	
Respondent’s history of mental health					
History of mental health problem					
Yes	8.5 (33)	28	84.8	(68.0, 93.7)	0.069
No	91.5 (357)	255	71.4	(66.5, 75.9)	
Disease for which currently received treatment					
None	82.3 (321)	230	71.6	(66.4, 76.3)	
Chronic NCDs	4.4 (17)	11	64.7	(39.6, 83.7)	
Pregnant mother	1.8 (7)	7	100.0	-	0.256
Lung diseases	7.7 (30)	25	83.3	(65.2, 93.0)	
Other infectious diseases	3.9 (15)	10	66.7	(39.6, 85.9)	
The psychological attributes of COVID 19 pandemic					
Tensed level being infected by COVID-19					
Severe (4, 5)	187 (48.0)	150	80.2	(73.8, 85.3)	
Moderate (2, 3)	166 (42.5)	117	70.5	(63.1, 77.0)	<0.001
No/minimal (0–1)	37 (9.5)	16	43.2	(28.2, 59.6)	
Tensed level of a family member being infected by COVID-19					
Severe (4, 5)	309 (79.2)	229	74.1	(68.9, 78.7)	
Moderate (2, 3)	67 (17.2)	46	68.7	(56.5, 78.7)	0.278
No/minimal (0–1)	14 (3.6)	8	57.1	(30.7, 80.1)	
Working hour (daily) before this pandemic					
≤6	31.8 (124)	96	77.4	(69.2, 84.0)	
7–10	51.5 (201)	145	72.1	(65.5, 77.9)	0.121
11–14	13.6 (53)	32	60.4	(46.6, 72.7)	
≥15	3.1 (12)	10	83.3	(50.5, 96.1)	
Having sleeping disturbances in the last weeks					
Never	14.9 (58)	28	48.3	(35.7, 61.1)	
Occasionally	26.9 (105)	65	61.9	(52.2, 70.7)	
Sometimes	25.9 (101)	76	75.2	(65.8, 82.7)	<0.001
Often	22.0 (86)	76	88.4	(79.6, 93.7)	
Always	10.3 (40)	38	95.0	(81.8, 98.8)	
Average sleeping hours in the last 4 weeks					
≤ 4	3.9 (15)	14	93.3	(63.0, 99.1)	
4–6	28.2 (110)	88	80.0	(71.4, 86.5)	
6–8	42.8 (167)	113	67.7	(60.2, 74.4)	0.051
8–10	21.8 (85)	58	68.2	(57.5, 77.3)	
≥ 10	3.3 (13)	10	76.9	(46.4, 92.8)	
Agitated more quickly than usually					
Yes	41.3(161)	144	89.4	(83.6, 93.4)	
No	35.6(139)	74	53.2	(44.9, 61.4)	<0.001
May be	23.1(90)	65	72.2	(62, 80.5)	
Agitation about human contact					
I am very agitated	10.3 (40)	37	92.5	(78.9, 97.6)	
I am agitated, but I tolerate	44.9 (175)	138	78.9	(72.1, 84.3)	
No change, like before	36.9 (144)	86	59.7	(51.5, 67.5)	<0.001
Better than before	6.4 (25)	18	72.0	(51.3, 86.3)	
Way better than before	1.5 (6)	4	66.7	(23.6, 92.8)	
Community impact of COVID 19 pandemic					
Facing any obstacles/humiliation while getting into or back from work by regulatory forces					
Yes	17.2 (67)	48	71.6	(66.4, 76.8)	
No	73.8 (288)	207	71.9	(59.6, 81.2)	0.586
I do not remember	9.0 (35)	28	80.0	(63.3, 90.3)	
Fear of getting assaulted /humiliated on your way to hospital or home					
Yes, by regulatory forces	21.0 (82)	65	79.3	(69.0, 86.8)	
Yes, by general people	16.7 (65)	52	80.0	(68.4, 88.1)	
Both 1 and 2	4.1(16)	11	68.7	(42.4, 86.8)	0.031
Not at all	51.0 (199)	131	65.8	(58.9, 72.1)	
No response	7.2 (28)	24	85.7	(67.1, 94.6)	

Of all respondents, more than 80% reported they had no symptoms of COVID-19, were unexposed to COVID-19, and had access to PPE, either from self-funded or sponsored sources. Although more than half of the physicians reported that they were advised to abstain from media and social media, a similar number indicated that they were using social media even more during the pandemic that before. Regarding psychological impact of the COVID-19 the majority of participants (more than 90%) were worried about themselves or a family member becoming infected by COVID-19. Almost 85% of the physicians were facing some disturbances to sleep and around 55% were feeling agitated by human interaction.

### PHQ-9 Score

**Figure 1:**
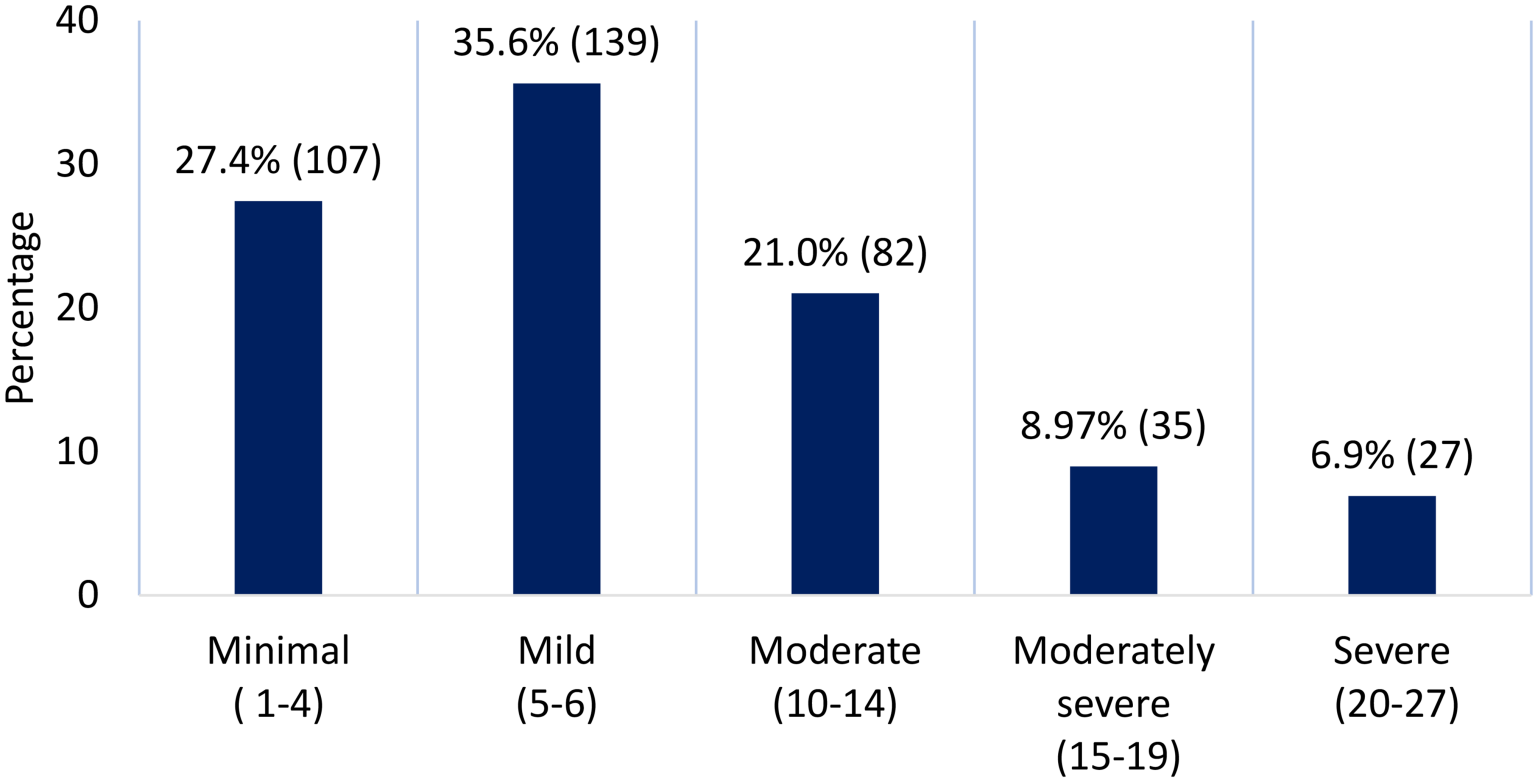
Depression symptoms severity level during COVID-19 pandemic, measured using PHQ-9, was determined in 390 physicians.

The sample mean of PHQ-9 score was 8.6 (standard deviation 6.0). Among all the respondents, 144 (36.9%) had a moderate or severe depression (score > 10), 139 (35.6%) rated mild depression (score 5–6); and 170 (27.5%) had minimal depression (score 1–4; see Figure 1 for a full breakdown of the data in each category).

### Predictors of Depressive Symptoms Among Physicians

Adjusted stepwise binary logistic regression indicated that physicians’ gender, marital status, source of PPE, advice to abstain from media, the fear of being infected by COVID-19, and having trouble sleeping (in the last week) were significantly associated with the presence of depression (Table 2). Female physicians were 1.9 times more likely to suffer from depression in the COVID-19 pandemic. Similarly, physicians who received advice to avoid media in the pandemic situation were more likely to have depression compared to those who were not. Depression was higher among moderately tensed physicians (AOR = 6.2; 95% CI: 2.1, 18.1) and those who had sleeping problems (AOR = 14.2; 95% CI: 2.8, 79.7) compared to those who had no such issues. Contrarily, married physicians were 60% less likely to experience depression compared to unmarried physicians. Similarly, physicians who received PPE both from the government of Bangladesh (GOB) and hospital were 70% less chance to be depressed compared to those who self-funded their PPE.

**Table 2:** Logistic regression analysis of depression measured by PHQ-9 with physicians’ different attributes, 2020 Bangladesh (*N* = 390).

Variables	Depression			
	UOR (95% CI)	*p*-value	AOR (95% CI)	*p*-value
Socio-demographic characteristics				
Age in year				
24–27	1.8(0.6–5.1)	0.269		
28–31	1.2(0.4–3.6)	0.729		
≥32	0.8(0.3–2.7)	0.768		
≤23	Reference			
Gender				
Female	2.5(1.6–3.9)	<0.001	1.9(1.1–3.5)	0.026
Male	Reference			
Marital status				
Married	0.5(0.3–0.9)	0.009	0.4(0.2–0.8)	0.006
Divorced/separate				
Unmarried	Reference			
Household related information				
Having sufficient earning				
Yes	0.3(0.2–0.6)	<0.001		
Not sure	0.3(0.2–0.7)	0.002		
No	Reference			
Personal protective equipment (PPE)				
Source of PPE				
Government	0.5(0.2–1.4)	0.170	0.5(0.1–1.7)	0.273
Hospital	0.6(0.3–1.1)	0.121	0.6(0.3–1.3)	0.197
Both Gob and hospital	0.4(0.2–0.8)	0.006	0.3(0.1–0.8)	0.014
Local people/NGOs/sponsored	0.4(0.2–0.8)	0.014	0.5(0.2–1.2)	0.132
Self-funded	Reference			
Exposed to media				
Got information to stay away from media				
Yes	3.5(2.1–5.8)	<0.001	2.8(1.5–5.3)	0.002
Not sure	1.8(0.9–3.4)	0.092	1.5(0.6–3.6)	0.366
No	Reference			
The psychological attributes of COVID 19 pandemic				
Tensed level being infected by COVID-19				
Severe (4, 5)	3.1(1.5–6.5)	0.002	3.4(1.2–9.7)	0.023
Moderate (2, 3)	5.3(2.5–11.2)	<0.001	6.2(2.1–18.1)	0.001
No/minimal (0–1)	Reference			
Having sleeping disturbances in the last weeks				
Occasionally	1.7(0.9–3.3)	0.094	1.7(0.8–4.0)	0.194
Sometimes	3.3(1.6–6.5)	0.001	2.9(1.2–7.0)	0.016
Often	8.1(3.5–18.8)	<0.001	4.6(1.6–13.1)	0.004
Always	20.4(4.5–92.4)	<0.001	14.9(2.8–79.7)	0.002
Never	Reference			
Agitated more quickly than usually				
Yes	7.4(4.1–13.6)	<0.001		
May be	2.3(1.3–4)	0.004		
No	Reference			
Agitation about human contact				
I am very agitated	8.3(2.4–28.3)	0.001		
I am agitated, but I tolerate	2.5(1.5–4.1)	<0.001		
Better than before	1.7(0.7–4.4)	0.248		
Way better than before	1.3(0.2–7.6)	0.735		
No change, like before	Reference			
Community impact of COVID 19 pandemic				
Fear of getting assaulted /humiliated on your way to hospital or home				
Yes, by regulatory forces	2.0(1.1–3.6)	0.027		
Yes, by general people	2.1(1.1–4.1)	0.034		
Both 1 and 2	1.1(0.4–3.4)	0.812		
No response	3.1(1–9.3)	0.043		
Not at all	Reference			

*Unadjusted odds ratio (UOR) shows the odds ratio of the individual predictor variable on the prevalence of depression. Adjusted odds ratio (AOR) shows the effect of all influential confounding variables on the prevalence of depression*.

## Discussion

This cross-sectional study investigated for depressive symptoms, and the predictive factors which might be responsible for initiating depressive symptoms, among physicians during the COVID-19 pandemic in Bangladesh. The findings of this study show that 72.6% physicians were suffering from mild to severe depressive symptoms, with PHQ-9 scores ranging from 5 to 27. This prevalence, in Bangladesh, is significantly higher than documented prevalence of depressive symptoms within other countries all of which have comparably used the PHQ-9 as the primary measuring tool for depressive symptom. For example, in Wuhan, China a study found a 13.5% prevalence among 5,062 physicians, nurses and clinical technicians in all clinical departments (Zhu et al., 2022). A nationwide observational study in India among 350 physicians and nurses who were engaged in triaging, screening, diagnosing, and treating COVID-19 patients found an 11.4% prevalence (Wilson et al., 2020). Our findings are closer to those reported by Lai et al. (2020), who found moderate to severe depressive symptoms in 50.4% of 1,257 nurses, physicians and other front liners in multiple regions of China (Lai et al., 2020). The findings of our study suggest that there are a number of predictors associated with the prevalence of depressive symptoms among physicians in Bangladesh. For instance, being in the age group 24–27, being female, being unmarried, not having sufficient earnings, lacking sufficient food supply at home, having symptoms of COVID-19, not getting enough PPE as well as self-funding for PPE, constant checking of news and having difficulty in staying away from the media, and finally having previous history of mental health issues are important predictors to develop depression symptoms. In relation to gender, our findings are in line with Pappa et al. (2020), who stated that women are at a disadvantage of suffering more from depression, which might worsen during the pandemic (Pappa et al., 2020). Elbay et al. (2020) in their study also stated being female is one of the prominent risk factors of suffering from depression (Elbay et al., 2020). Fino et al. (2021) reported significantly higher levels of distress and sleep–wake difficulty among female frontline healthcare workers compared to male frontline healthcare workers in Italy. Another report by Fino et al. (2021) indicated that the exposure to dying COVID-19 patients and the suffering of family members during the pandemic have had a greater impact on the mental health of female physicians. A recently published study by Hasan et al. (2022) conducted in Bangladesh acknowledged the existing gender gap for experiencing more anxious and depressive symptoms among women than men reporting a higher prevalence of psychological distress among women physicians. These findings can be explained by the report published by Fino et al. (2019) where they presented the modulating role of gender and aggression in emotional reactions of nursing students (Fino et al., 2019). The higher emotional reactivity to highly aversive situations might be the outcome of exposure of female health workers during the pandemic who have higher vulnerability to distress compared to males.

Our finding that physicians’ overuse of social and print media during the pandemic was a significant factor in developing depressive symptoms is in line with the findings of Ahmad and Murad (2020), who discuss how the spread of COVID-19 related fear and panic, particularly through social media, had a significant negative impact on psychosocial wellbeing (Ahmad and Murad, 2020). Furthermore, it has been well established that information transmitted *via* social media might be not applicable for certain geographical areas, or may even in some cases be incorrect or ‘fake news,’ which can ultimately cause depression, stress and anxiety among individuals irrespective of their history of mental illness (Gonzalez-Padilla and Tortolero-Blanco, 2020). In our study, from adjusted and unadjusted regression analysis, it was found that physicians who were suffering from sleep problems had a higher level of depressive symptoms compared to those who never suffered from sleep problems. Liang et al. (2020) have stated that the prevalence of sleep disturbance has increased during the pandemic when compared to any other time (Liang et al., 2020). Abdulah and Musa (2020) also reported that, among physicians exposed to COVID-19, the prevalence of sleep disturbance was 68.3%, while the prevalence was 45.5% in a similar study in the same region in 2019 (Abdulah and Musa, 2020). Sahin et al. (2020) stated that health care workers experience traumatic events within their working lives, for example, patients dying alone and having to break this news to the family members (Sahin et al., 2020). These experiences can cause the development of depressive symptoms as well as causing burnout. Findings from studies conducted during the SARS and Ebola epidemics echo this statement (Liu et al., 2012; Jalloh et al., 2018).

We found that physicians in our study who were moderately tensed about being infected by COVID-19, were found to be suffering more from depressive symptoms than those who were not tense about being infected. Sahin et al. (2020) in their study stated that the death of a colleague, or a colleague being quarantined, was associated with depressive symptoms among health care workers (Sahin et al., 2020). Unadjusted odds ratio of our study showed that physicians who reported to be agitated, and agitated more quickly than before, had higher levels of depressive symptoms than their counterparts. The fear of being assaulted by regulatory forces and the general public were also seen to be factors related to the development of depressive symptoms. Perceived discrimination against people who have been suffering from COVID-19 as well as those who have been serving the patients during the pandemic is a common social behavior and can cause serious psychological problems (Liu et al., 2020). As a consequence of this, physicians are in fear of being assaulted or discriminated against due to perceived social stigma about being affected by COVID-19 as a service provider, which directly affect their mental health. Previous studies have emphasized alarmingly high rates of ‘physician burnout,’ characterized by emotional exhaustion, depersonalization, and low personal accomplishments (West et al., 2018), and alternatively increased risk of suicidal ideation and suicidal attempts in physicians (Rothenberger, 2017). Moreover, Montemurro reported suicides in India and Italy during this pandemic, as physicians experienced helplessness, acute psychological stress, and utmost fear of dying (Montemurro, 2020).

In a resource constrained country like Bangladesh, it is quite unusual to receive interventions for physicians’ mental wellbeing amidst the pandemic, where the health system has already been overloaded with the burden of both communicable and non-communicable diseases. The increasing prevalence of depressive symptoms among physicians is worsening the situation. In a setting where physicians are constantly concerned about PPE funding and effectiveness, the mere thought of mental health interventions seems nothing but a luxury. But this is a matter which cannot be overlooked. For this, proper initiatives including digital interventions should be undertaken as soon as possible. In this context, our study should act as a driver to government and clinical organizations, to ensure that they are concentrating on the mental health issues of physicians during the pandemic addressing gender specific needs. This study has the potential inform policy development regarding much needed approaches to lowering rates of depression in physicians in Bangladesh and build on the existing global body of evidence that this is an issue worth considering worldwide.

## Strengths and Limitations

This study provides concerning findings on the prevalence of depressive symptoms among Bangladeshi physicians during the COVID-19 pandemic; however, data should be interpreted carefully in light of specific limitations. The cross-sectional nature of the study design could not establish any causal relationships. Also, this study was carried out by conducting a web-based survey, which might generate sampling bias by excluding the physicians who do not have access to internet or are simply inactive in social media, and thus limit the generalizability of the findings. Self-reported responses on anxiety and depression symptoms only provide subjective data which may greatly differ from objective data, leading to response bias. Finally, although we tried to address major risk factors, several relevant variables, such as residence status (urban or rural), having children, domestic violence, moral dilemma associated with managing complex patients and information on physician’s work hours or perceived workloads were not included in the survey.

Despite these limitations, the study has several clear public health implications. Our results demonstrate the vulnerability of Bangladeshi physicians to develop depressive symptoms during the pandemic which should be closely monitored. Effective interventions to support gender specific needs should be designed to support physicians especially from low resource settings. We are also aware that the second wave of the pandemic was deadlier than the first one in the country. The first wave was a unique situation not only to the physicians or general people of Bangladesh but also to the people of the world. Besides, people went through series of lockdowns, closure of educational institutions, price hike, panic buying and many ‘newer’ phenomena during that period, let alone the physicians. The physicians had to go through unique challenges adapting to the ‘new normal’ with overwhelming work pressures. This study was conducted during such a critical period which was absolutely uncertain & a shock to everyone where the physicians faced additional challenges with extreme limited resources. We wanted to find out the actual mental health state of the physicians with a single focus on developing depression symptoms specifically mentioning the study period to give a context to the policy makers to keep this situation in mind and design appropriate interventions to support the physician to protect their mental health during any public health emergencies like this. Therefore, the gravity of this study is immense.

## Conclusion

Given the importance of the risk factors associated with physician’s depression symptoms identified in this study, urgent provision of adequate safety measures proper training before deployment in the isolation ward, additional incentives, and on-going monitoring and remote psychological support using digital technologies may aid in reducing physician’s psychological strain amid such a terrible pandemic and any future public health crisis like this. Specific mental health support needs of the female physicians should be addressed adequately and strategically.

## Data Availability Statement

The raw data supporting the conclusions of this article will be made available by the authors, without undue reservation.

## Ethics Statement

The studies involving human participants were reviewed and approved by Ethical Review Committee, Shaheed Suhrawardy Medical College, Dhaka, Bangladesh (Ref. no: ShSMC/Ethical/2020/12). The patients/participants provided their written informed consent to participate in this study.

## Author Contributions

MH, SFA, MR, VP, SQA, and FA: conceptualization. MH, SFA, MR, VP, DA, and RN: investigation. MH, SH, KK, and MB: methodology. MB: data analysis. MH, FS, SFA, and SH: resources. SQA and FA: supervision. AA, MB, SIA, MH, and SH: writing—original draft. AA, MH, MB, SIA, SH, FS, KK, SFA, MR, VP, TT, RN, DA, FA, SQA, HA, and RM: writing—review and editing. All authors contributed to the article and approved the submitted version.

## Conflict of Interest

The authors declare that the research was conducted in the absence of any commercial or financial relationships that could be construed as a potential conflict of interest.

## Publisher’s Note

All claims expressed in this article are solely those of the authors and do not necessarily represent those of their affiliated organizations, or those of the publisher, the editors and the reviewers. Any product that may be evaluated in this article, or claim that may be made by its manufacturer, is not guaranteed or endorsed by the publisher.
